# Supplementary material for: Are carbon emissions trading and green financial instruments synergistic? -Comprehensive quantitative research based on content analysis
Source: PLoS One. 2024 Mar 7;19(3):e0298601. doi: 10.1371/journal.pone.0298601 (PMC10919601; doi:10.1371/journal.pone.0298601)
Supplement: S1 Data — (ZIP) [file pone.0298601.s001.zip › Data/地级市Z_F工作报告.zip!/╣π╬≈/╟╒╓▌╩╨/└┤╫╘╣1⁄2╓┌║┼í╛┬φ┐╦╩2╛▌═°í┐_2013.docx]

各位代表：

现在，我代表市人民政府向大会报告工作，请予审议，并请各位政协委员和其他列席人员提出意见。

一、2012年工作回顾

2012年，我市经受住了复杂多变的宏观经济环境的严峻考验，经济发展在前年实现“撑杆跳”后，继续保持了平稳较快增长。在市委的坚强领导下，全市人民坚决贯彻中央和自治区的决策部署，坚持稳中求进、好中求快、多出成效的工作基调，大力弘扬新时期钦州精神，解放思想，创新实干，攻坚克难，强化执行，较好地完成了市四届人大二次会议确定的各项任务，经济社会发展亮点纷呈，呈现出平稳协调发展的良好态势。

——中马钦州产业园区正式开园，川桂临海产业园落户钦州，自治区级高新区获批设立，中国石油化工（钦州）园区获批为首批国家级循环化改造试点园区，对内对外全方位开放的格局进一步形成；

——亿吨大港建设稳步推进，30万吨级主航道竣工，30万吨级油码头完成水工部分，一批深水码头相继建成并投入使用，铁路、高速公路加快建设，港口集疏运体系基本完善，对经济的拉动作用进一步增强；

——千亿元石化产业集群加速形成，造纸、粮油等六个百亿元产业快速发展，中船大型海工修造及保障基地、澄星磷化工基地等重大项目开工建设，县域工业发展实现重大突破；梦之岛、沃尔玛、大地影院等一批国内外知名连锁品牌入驻，现代服务业发展初具规模，产业竞争力明显提高；

——滨海新城建设展现新形象，扬帆大道南延长线基本贯通，北部湾大道一期建成通车，南北高速公路茅尾海出口、北部湾钻石海岸国际海鲜城、和谐塔公园等一批功能性项目相继建成，中心城区的辐射带动功能开始显现；

——社会建设步伐加快，开工建设北部湾大学和千年古陶城，成功举办“两节一赛”，荣获“中国坭兴陶之都”称号；科学调整城区行政区划，创新社会管理体制，全力办好为民办实事“十项工程”，人民生活水平进一步提高。

这些成绩的取得，标志着我市推进“五年跨越、十年巨变”取得重要阶段性成果，经济社会发展的基础更加扎实，体制更加完善，前景更加辉煌。一年来，我们主要做了以下工作。

（一）坚定不移保增长，经济运行质量有了新提高。经济总量继续扩大，实现地区生产总值724.5亿元，增长12%，人均gdp由2011年的20896元提高到23202元；规模以上工业总产值突破千亿元，达到1093亿元，增长16.4%；海洋经济总产值突破200亿元，增长13.6%。经济发展质量稳步提高，财政收入139.2亿元，增长13.1％，一般预算收入33.6亿元，增长31.3%，税收收入比重达89.5%。银行业金融机构本外币存款余额622.7亿元，本外币贷款余额447.3亿元，比2011年分别增加88.7亿元和69.5亿元。城镇居民人均可支配收入21600元，增长12.2%；农民人均纯收入7140元，增长15.8%。扩大内需成效明显，完成全社会固定资产投资652.6亿元，增长16.9%；全社会消费品零售总额237.6亿元，增长16.3%。外经贸易逆势增长，外贸进出口总额37.7亿美元，增长26.1%；进口额超1000万美元的企业达到25家。招商引资力度加大，引进国内到位资金550亿元，增长25.3%；实际利用外资4.7亿美元，增长15.2%，总量位居全区第三位。

（二）千方百计促转型，产业升级迈出新步伐。坚持在产业培育的过程中推进产业转型，促进三次产业融合发展，荣获广西工业产业发展一等奖。千百亿元产业培育实现重大突破，临港工业加快集聚。中船项目启动建设，结束了广西沿海无现代化大型修造船企业的历史；汽车整车生产项目进展顺利。石化产业集群加快发展，中石油含硫原油加工配套工程全面进入设备安装；建成天恒化工、泰兴石化项目，开工建设澄星磷化工等项目。百亿元产业加速崛起，清华同方、中粮二期等建成投产，锐丰钒钛铁、华汇镍业、中电投临时供热工程、汇海粮油加工等项目加快推进。“县域工业突破工程”三年目标基本实现，县域规模以上工业企业达到231家，总产值达328.1亿元，增长63.8%，对全市规模以上工业增长贡献率达到78.4%。灵山县、浦北县、钦南区的地区生产总值、工业总产值以及各县区全社会固定资产投资均突破100亿元。县域工业园区基础设施进一步完善，乡镇工业集中区初步形成规模集聚效应。农业特色优势产业加快发展，荔枝、香蕉等种植和加工业全面提升，大蚝、对虾、名贵龟鳖、奶水牛等特色养殖和林下经济加快发展。钦南台湾农民创业园、 “中国龟谷”启动建设。新增农业产业化重点龙头企业14家、农民合作社117家。农产品安全监管服务机构率先在全区实现市、县、镇、村四级全覆盖。灵山县获评为“中国养蛇之乡”，灵山荔枝、灵山凉粉、浦北香蕉、钦南区海鸭蛋获国家地理标志产品称号，“宇峰”荣获中国驰名商标。粮食播种面积、总产量实现双增。现代服务业实现提速升级，年年丰、恒基金湾等商业广场建成使用，一批国内外商业品牌运营良好，新型商业业态不断涌现，全市规模连锁经营企业发展到30多家，门店1950多个。节庆经济蓬勃发展，成功举办2012钦州国际蚝情节、国际陶艺文化节暨第四届全国陶瓷技艺大赛，三娘湾观潮节获评广西十大节庆品牌。全年接待游客692.7万人次，实现旅游收入51亿元。交通运输业快速发展，公路和水路客货运周转量分别增长27.2%和49.5%。金融创新成效显著，跨境贸易人民币结算业务突破10亿元大关。全年政府性融资到位资金89亿元，金融产品融资到位59.6亿元，银行贷款融资到位29.5亿元，均比2011年大幅度增加。以环境倒逼机制推动产业转型升级，万元gdp能耗约下降2%，化学需氧量、二氧化硫控制在年度排放总量控制目标内，超额完成年度节能减排目标任务。环境质量持续改善，城镇饮用水源地水质达标率及空气质量优良率均达100%。

（三）坚持港城联动发展，城乡面貌发生新变化。着力推进亿吨大港建设，30万吨级主航道、大榄坪12#—13#泊位等港口标志性工程相继竣工，国投煤炭码头、大榄坪1#—6#泊位、钦州港三期工程加快建设，港口吞吐能力达到8565万吨。港口集疏运体系加快完善，南宁—钦州—防城港高铁开始联调联试，钦州—北海高铁基本完成铺轨。钦州—崇左高速公路通车，六景—钦州港高速公路完成路面工程。积极拓展航运物流市场，新开通钦州港—宁波—连云港—日照—青岛直航航线、钦州保税港区—大连北良港的粮食运输航线、钦州港—云南昆明的“五定”班列等，海铁联运到发箱量增长21%。港口吞吐量5622万吨，增长19.3%，其中集装箱吞吐量47.4万标箱，增长17.9%，继续位居北部湾港口群第一位。坚持统筹城乡协调发展，全市城镇化率达到41%。滨海新城建设大框架形成，白石湖、沙井岛、辣椒槌三大片区交通基础设施初步完善，白石湖公园、东盟生态园、茂盛安置小区等加快建设。中心城区和重点城镇建设展现新面貌，完成城市总体规划和市县镇三级土地利用规划修编，编制完成高新区总体规划。中心城区一批市政基础设施项目竣工或加快推进。开展县镇村道路通畅三年大会战，初步形成中心城区辐射各县区、镇、村的四级路网结构。加快建设各具特色的县城和重点镇，完成重点村镇“五个一工程”和“两延伸”工程年度任务。启动“镇改办、村改居”试点工作。深入推进“园林生活十年计划”，加快创建国家园林城市，中心城区建成区绿化覆盖率达34.6%，绿地率29.7%，人均公园绿地面积7.6平方米。全面完成“绿满八桂”造林绿化工程和海防林、退耕还林配套荒山造林工程，森林覆盖率达52.9%。启动建设茅尾海综合整治项目一期工程。积极实施“城乡清洁工程”，加强城乡风貌改造，完善与居民生活密切相关的配套设施，主城区垃圾无害化处理率达95%，市政道路装灯率达100%，农村环境连片综合整治成效初显。完成可再生能源建筑应用示范城市建设任务。加大灌区节水改造、病险水库除险加固、标准海堤、农村饮水安全、沼气池等农业和农村基础设施建设力度，农村公共服务能力进一步增强。

（四）注重开放扩合作，改革创新增添新活力。国务院批准设立中马钦州产业园区，两国总理共同参加园区开园仪式；两国政府签署马中关丹产业园区协定，首创了“两国双园”的国际合作新模式。与四川共建川桂临海产业园，西南出海大通道作用日渐实现。着力放大开发开放平台效应，保税港区国际酒类交易中心、进口汽车检验中心等项目竣工，成功开展汽车、红酒进口等特色业务，国家质检总局批准筹建全国进口酒类综合服务产业知名品牌示范区；钦州港经济技术开发区荣膺“中国最具投资吸引力园区”；中国石油化工（钦州）园区成为首批国家级循环化改造示范试点园区之一。深化国际交流合作，积极探讨与东盟实现海上互联互通，与英国圣海伦斯市正式缔结国际友城关系，同韩国土地公社确定共建中韩绿色智能新城框架协议。切实加强招商引资工作，探索建立重大项目帮办服务制度，与中船、中国通用、中交等大型央企签订了项目合作框架协议。着力提升科技创新能力，全市专利申请量410件、增长94.3%，专利授权量173件，认定同方数字图传及节能技术、现代中药制药等4家市级工程技术研究中心，建成高新技术产业孵化基地和大学生创业孵化基地，新增高新技术企业9家。理顺市与城区权责关系，完成钦南区、钦北区行政区域调整，实行城市管理中心下移，下放征地搬迁、环卫等管理权限。实施土地网上交易，推行产业园区节约集约用地。开展无居民海岛开发利用保护规划编制，率先在广西开展海域使用权招拍挂试点。开展“尾雁赶队”活动，抓好行政效能再提速和扩权强县工作，整体投资环境和投资软环境调查评比连续两年名列全区首位，第四次荣膺“中国十佳投资环境城市”。

（五）以人为本惠民生，社会发展取得新进步。坚持政府投资向民生领域倾斜，全年共投入38.7亿元，基本完成10项为民办实事工程，解决了一批事关群众切身利益以及社会和谐稳定的实际问题。发放城乡低保资金2亿多元。开工建设保障性住房9628套，新增发放廉租住房租赁补贴1073户；建成保障性住房6196套，完成农村危旧房改造9008户；调整廉租住房实物配租对象收入标准，月人均增加64元。继续实施“三业”行动富民工程，突出推进“万千百行动计划”，扶持800多家微型企业成功创业。城镇新增就业2.5万人、农村劳动力转移就业新增7.2万人；城镇登记失业率3.5%，低于年度控制目标。全市就业工作荣获国务院嘉奖。社会保障体系不断完善，率先在全区整合社会保险经办机构，城乡居民养老保险制度实现全覆盖，参保人数达到96.4万人。最低生活保障制度实现全覆盖。初步建成市级卫生信息平台，新农合在全市范围内实现即时结报，新农合参保率达到96.9%。建成五保新村82个，为63个敬老院、708个五保村配备了管理人员。突出抓好社会管理创新，“平安和谐家园”创建活动不断深化，领导干部接访、带案下访和包案制度有效落实，“天网工程”加快推进。落实安全生产“一岗双责”责任制，强化重点领域安全监管，安全生产形势保持稳定。食品药品监管不断加强。应急管理体系进一步完善，国防动员、民兵预备役、优抚安置和法律援助工作稳步推进，争创全国双拥模范城正式启动。各级各类教育水平稳步提升，引进建设广西财贸职业学院，建成市职教中心二期、市第十一小学、市实验幼儿园、市二中附属幼儿园及85个中小学校舍安全工程，改扩建469所镇村幼儿园。建成89个村级公共服务中心和221家农家书屋。人才工作扎实推进，成立留学人才创业园，制定出台“1+4”人才政策，启动实施“三百优才”培养计划。扎实开展广播电视村村通和公益电影、校园电影、园区电影放映工作，提前三年完成城市影院数字化改造任务。文化体制改革成效明显，院团改制全面完成，成立了非物质文化遗产传承保护中心和白海豚演艺有限公司，全市“三馆一站”实现免费开放。建成市第一人民医院综合楼，市中医医院迁建项目加快推进。防治艾滋病攻坚工程成效显著。全民健身活动广泛开展。全面推进诚信计生，低生育水平持续稳定。残疾人“五个一”帮扶工程全面完成，镇级工作网络实现全覆盖。质量兴市战略深入实施。海关、出入境检验检疫、海事、边检、口岸等在我市对外开放中发挥了重要作用；税务、人民银行、银监、工商、供电、烟草、通信、邮政、盐务等在服务地方发展中作出了积极贡献；体育、民族、宗教、外事、侨务、统计、农机、供销、气象、地震、水文、人防、海防、新闻出版、科普、档案、地方志、妇女儿童、老龄、残疾人等各项事业都取得了可喜进展。

一年来，我们深入贯彻落实科学发展观，认真履行人民赋予的权力，自觉接受市人大及其常委会的法律监督、工作监督和市政协的民主监督，主动听取人大代表、政协委员的意见和建议，认真办结人大代表议案和政协委员提案。支持人民法院、人民检察院依法公正独立行使审判权、检察权。深入开展“制度建设年”和“好干部、善执行”活动，切实加强政府自身建设，大力推进政府职能转变，完善民主决策机制，全面推进依法行政，努力提高行政效能。在工作中坚持勤政廉政，强化行政监察和审计监督，推进“审计整改年”活动，全力以赴推动各项工作的贯彻落实。

各位代表，过去一年的成绩来之不易，这是自治区党委、自治区人民政府和市委正确领导的结果，是市人大、市政协有效监督和大力支持的结果，是全市人民凝心聚力、奋力拼搏的结果。在此，我代表市人民政府，向全市人民和外来建设者，向人大代表、政协委员，向各民主党派、工商联、各人民团体和社会各界人士，向驻钦各单位、军警部队官兵，向关心和支持钦州发展的海内外朋友们，表示衷心的感谢！

我们也要清醒地看到，我市经济社会发展还存在不少困难和问题。一是经济仍然处于艰难的爬坡阶段，地方可用财力不足，资金短缺、征地搬迁等仍是制约经济发展的主要瓶颈；二是产业发展不平衡，多数产业尚处于培育期，产业配套还不完善，新增长点不多，经济增长存在下行压力；三是城乡发展不够协调，县域工业刚刚起步，县区基础设施建设比较滞后，农业农村发展需要更多倾斜；四是社会建设还存在不少薄弱环节，生态环境、交通秩序和城市卫生需要花大力气整治，维护社会稳定压力继续增大；五是政府自身建设和管理有待加强，干部的执行力和创新能力还需要进一步提高。对于这些问题，我们将高度重视，认真加以研究，采取有力措施，切实加以解决。

二、2013年工作安排

2013年是全面贯彻落实党的十八大精神的开局之年，是加快推进“五年跨越、十年巨变”的关键一年，是为提前全面建成小康社会奠定坚实基础的重要一年，做好今年的工作意义重大。

今年政府工作的总体要求是：全面贯彻落实党的十八大和中央、自治区经济工作会议精神，坚持以邓小平理论、“三个代表”重要思想、科学发展观为指导，紧紧围绕主题主线，深入实施“建大港、兴产业、造新城、强科教、惠民生”发展方略，牢牢把握“稳中求进、开拓创新、凸显成效”的工作基调，继续深化改革创新，充分发挥开放合作的引领作用，大力推进新型工业化、城镇化，切实提升服务业，扎实培育可用财力，着力保障和改善民生，保持物价总水平基本稳定，提高城乡居民收入，维护社会和谐稳定，为提前全面建成小康社会打下坚实基础。

经济社会发展的主要预期目标是：生产总值增长12%；公共财政预算收入增长15%；全社会固定资产投资增长21%；规模以上工业总产值增长20%；港口吞吐量6200万吨，其中集装箱60万标箱；外贸进出口总额增长10%；实际利用外资增长5%，引进国内到位资金增长10%；社会消费品零售总额增长15%；城镇居民人均可支配收入增长13%，农民人均纯收入增长14%；居民消费价格指数涨幅控制在4%左右；万元生产总值能耗下降2.2%，化学需氧量、二氧化硫、氨氮化物和氮氧化物排放总量控制在自治区下达的目标之内；城镇登记失业率4.2%以内，城镇化率达到44%左右，人口自然增长率控制在10‰以内。

各位代表，上述目标经过科学测算，是积极进取、符合实际、切实可行的，必须确保全面实现。围绕实现上述目标，今年政府工作要着力在以下八个方面实现突破。

（一）以实施五大工程为重点，在加快工业化进程上求突破。以新型工业化为导向，着力推动产业集中集约集聚发展，不断提升产业发展层次和综合竞争力。

大力实施“千百亿产业崛起工程”。制定实施千百亿产业三年攻坚计划，加快发展“2+6”现代产业体系，力争2015年产值达到1800亿元。积极争取国投燃煤电厂二期、中电投热电联供、中石油lng等一批重点项目获国家、自治区审批核准，争取汽车整车生产项目实现开工。全力推进中船大型海工修造及保障基地、港机重工、国投燃煤电厂二期等一批项目加快建设。力促中石油含硫原油加工配套工程、澄星磷化工、中电投临时供热工程、汇海百万吨大豆加工、华汇镍铁、锐丰钒钛铁等一批项目建成投产。尽快启动中石油二期、金桂二期等重大项目前期工作。

大力实施“县域工业壮大工程”。制定完善县域工业发展壮大的政策措施，力争2015年县域规模以上工业产值达到900亿元，占全市规模以上工业产值40%以上。推动各县区以申报自治区级经济技术开发区为载体，加大县区基础设施投入，完善园区基础设施和解决外部交通瓶颈制约，为县区工业经济发展提供有力支撑。支持钦南区进口资源及新材料加工区扩区，推进县区重点镇工业集中区建设，加快引进一批“短平快”中小项目入驻，打造县域特色工业新增长点。着力培育重点龙头企业，积极发展微型企业，抓好中小企业的技改、扩能增产。年内新增规模以上企业50家、亿元以上企业20家左右。

大力实施“高新产业培育工程”。坚持引进和培育相结合，着力推进电子信息、新能源、新材料、节能环保、生物技术等战略性新兴产业在我市布局和发展。积极构建国家级和自治区级研发平台，重点引进一批科技创新型企业，进一步壮大高新技术产业规模。加快构建政府和市场投入相结合的科技孵化体系，争取新引进孵化企业5-10家。继续实施企业“专利倍增计划”，力争高新技术企业突破20家，培育2家创新型试点企业，新增2-3家自治区级研发中心。完善高新技术产业政策服务平台，探索建立科技投融资体系，组建风险投资公司和担保公司。围绕推进产学研结合，加强科技资源整合,加快培育一批科技成果转化与产业化基地。

大力实施“园区基础设施大会战工程”。全力支持中马钦州产业园区开发建设，按照“打基础、活机制、出成效”的总要求，加快 “三纵三横”路网和给排水管网建设，力争年内20个产业项目签约入园、10个产业项目实质性开工。钦州保税港区要争取建成二三期路网及监管设施，并实现封关运作，积极推动入区经营性项目、企业生活配套区及加工产业标准厂房建设。高新技术产业开发区要积极探索市场化的投资体制，加快一二期路网和配套设施建设。钦南台湾农民创业园要编制完成园区总体规划和产业发展规划，研究制定园区管理机制和优惠政策，建设和完善园区道路、水利设施等基础设施，力争一批农业产业化项目入园。

大力实施“港口服务提升工程”。加快港口集疏运体系建设，力争新增吞吐能力2000万吨，港口吞吐能力超亿吨。开工建设30万吨级支航道、油码头栈桥等项目，力争建成30万吨级油码头。加快国投交通煤炭码头、金鼓江航道等一批深水航道码头建设。建成运营钦州港至保税港区铁路，开工建设钦州火车东站综合客运枢纽，整合提升鹰岭铁路专线。服务好龙门跨海大桥、沿海高速公路改扩建等项目建设。启动三墩作业区开发建设，扩大港口发展空间。完善钦州口岸硬软件设施，加快建设钦州港口岸联检大楼，开工建设钦州果子山边地贸口岸联检楼以及鹰岭、勒沟作业区围网等监管设施；创新推行口岸查验新模式和激励机制，推进电子口岸建设，降低物流成本。提升港航服务水平，重点培育发展商业、会展、金融、保险、船舶注册等港航服务。优化升级钦州港集装箱航线，扶持开通钦州港—台湾高雄、钦州港—天津—营口南北直航线，扩大内外贸同船运输和海铁联运业务，推动与西南内陆主要节点城市的“无水港”建设。

（二）以滨海新城建设为重点，在新型城镇化建设上求突破。坚持城市建设与管理并重，完善功能，提升品质，优化生态，全力推进新型城镇化，统筹城乡协调发展。

加快滨海新城规模化高端化建设。完善新城配套功能，增强区域发展的集聚辐射能力。推进交通、教育、文化、卫生、生态环境、市政设施等六大工程建设，推动白石湖、沙井岛、辣椒槌三大片区建设出新成效。建成扬帆大道南延长线、北部湾国际大酒店、东盟生态园、白石湖公园、规划展示馆、茂盛安置小区一期、风情酒吧街等，开工建设北部湾大道二期、奥博水上运动中心等项目，争取运动休闲、商贸旅游、文化娱乐、创意研发等一批功能性项目落户。

打造品质高尚、功能完善的城市中心区。建成蓬莱大道、环城东路等新区配套道路，加快建设一批上规模精品楼盘。加快高新区综合功能配套区建设，打造产业园区与城市新区融合发展、一体化发展的示范区。改造提升人民路等老街区，开展城中村改造试点。完善城区市政公用设施，鼓励民营资本投入公共停车场建设，开工建设金海湾东大街东段、钦江五桥、城市生活垃圾焚烧发电等项目。推进城乡公交一体化，扩大公交线路覆盖面。加快推进钦州港区行政商务中心一二期路网、保税港区联检部门办公生活区、农民回建房等项目建设。

着力提升城市管理水平。统筹推进国家卫生城市、国家园林城市创建工作。坚持实施“园林生活十年计划”，加强市区重要街区的城市设计和功能提升，丰富城市文化内涵，打造一批样板街、示范路和精品区，建设一批景观公园、节点绿化、街头绿地。大力开展市容环境综合整治，加大禁违拆违力度，整治道路抛洒、乱涂乱贴乱放等不良现象。规范配置停车场、街巷路灯、交通标识，下大力气改善主城区交通拥堵、违章行驶、占道经营等状况。深化拓展“城乡清洁工程”。完善数字化城市管理体系，切实提高城市管理效能。

推进县城及重点镇建设。实施新型城镇化发展战略，构建“一区三轴”城镇发展布局。加快建设灵山县新城片区、浦北县城金浦片区和钦南、钦北新城区，每个县区加快推动2个以上重点镇建设，争取一批镇、村列入自治区重点名镇名村建设。抓好灵山县创建广西经济强县、浦北县寨圩镇全国小城镇发展改革试点和“镇改办、村改居”试点工作。推进重点城镇路网、给排水、污水处理设施建设。继续实施镇村规划集中行动，力争完成所有镇总体规划和重点镇控规编制，加快村庄规划编制。加强新农村建设，大力整治村庄环境，改善农村人居环境。

（三）以扩大城乡消费为重点，在现代服务业提速升级上求突破。研究出台支持服务业发展的政策措施，加快培育消费增长点，推动经济增长由投资拉动逐步向投资与消费并重的转变。

科学布局城市商业网点。重点抓好河东新区、港区新城等商业网点建设，完善年年丰广场、恒基金湾广场等城市综合体，打造城市中心商业区。大润发、北京百分百、华润、现代新街市等项目建成营业，吸引国际著名商家、品牌旗舰店进驻。加强县区商业网点规划建设，突出抓好县城镇商贸服务业发展。改造东风市场等一批农贸市场，推进县区农超市场建设。

加快培育现代物流业。科学规划港口功能，加快建设油品、石化、煤炭、粮油等专业物流体系。以钦州保税港区为龙头，重点发展保税物流、集装箱物流、汽车港口物流等新型物流业。加快建设综合物流加工区、皇马物流园、浦北物流中心、灵山粮油加工配送基地，积极推进东盟商贸城、中国—东盟农产品大市场、国际汽车城、虎邱钢材市场等大型专业市场建设，建成可再生资源回收集散市场。

积极发展金融保险服务业。发挥面向东盟的产业和贸易合作优势，尽快启动北部湾金融贸易中心的研究和规划。鼓励发展金融、保险、信息业，加快引进信托租赁、金融租赁等新型金融服务机构，积极发展村镇银行和各类担保机构。拓展筹融资方式，与大型金融服务商建立战略合作关系，组织企业发行中小企业集合债、中期票据、短期融资券等。加强企业直接融资步伐，加快推动企业上市。扩大离岸金融、跨境贸易人民币结算业务，积极申报国家级跨境金融改革试验区。

（四）以农业增效农民增收为重点，在特色农业发展上求突破。认真贯彻中央1号文件精神，大力发展高效、生态农业，推进传统农业向现代农业、农业大市向农业强市转变。

继续推进“特色农业提升工程”。加快调整优化农业产业结构，推进荔枝、香蕉、奶水牛、大蚝、名贵龟鳖等特色农业标准化、规模化生产，支持建设一批国家级、自治区级标准果园，建设国家级大蚝原种场等水产畜牧养殖示范基地。落实战略性合作伙伴，全面推进“中国龟谷”建设。大力发展林下经济。

着力提升农业产业化经营。培育重点龙头企业，新增一批自治区级农业产业化龙头企业，农产品加工率争取达到41%。发展一批农民合作社，完善农业社会化服务体系。积极创建农产品品牌，积极申报“钦州黄瓜皮”等国家地理标志产品，新注册一批农产品商标，争创2个以上名优农产品。

加强农村基础设施建设。突出抓好农田水利建设，完成一批农业综合开发、农村饮水安全、病险水库除险加固、海河堤防、中小河流治理、水土保持综合治理等项目。扎实推进县镇村道路通畅三年大会战，修通百姓致富路，解决群众出行难问题，通行政村水泥路通村率达到95%。加大农村沼气池建设力度。开展贫困村整村推进扶贫攻坚，改善贫困村和移民新村生产生活条件。

多渠道增加农民收入。落实强农惠农政策，大力发展特色优势农业，增加农民经营性收入。鼓励土地承包经营权流转，提高农民财产性收入。加强农民技能培训，引导农民有序外出务工和就地就近转移就业，增加工资性收入。扶持发展乡镇中小企业、休闲农业、乡村旅游，增加农民非农经营收入。

（五）以深化重点领域和关键环节的改革开放为重点，在焕发体制机制活力上求突破。坚持以开放为先导，以创新为动力，努力破解经济社会发展难题，着力构建跨越发展新优势。

推进各项重点改革。加快制定出台鼓励民间投资的政策措施，放心、放手、放胆发展民营经济，促进中小企业和微型企业加快发展，引导民间资本参与国企和政府建设项目投资。创新滨海新城、高新技术产业开发区管理体制，在人才引进和资金运作上赋予更多自主管理权。深化城区管理体制改革，实行城市管理重心下移，实现由执法管理为主向管理与服务并重转变。完善政府投融资体制，加强政府投资平台的规范化运作，探索城市经营新模式，着力增强造血功能，积极化解金融风险。以优化财源结构为重点，实施“地方财源三年倍增计划”，进一步增强政府可用财力。稳步推进城乡建设用地增减挂钩试点，实施征转分离、区位调整等政策。深化扩权强县工作，促进“一服务两公开”向基层延伸。

培育区域合作新增长点。加大中马钦州产业园区、川桂临海产业园、中国石油化工（钦州）园区、高新技术产业开发区、滨海新城等重点园区和城区的招商引资力度，推动一批产业项目落地建设。重点引进世界500强、中国500强、知名上市公司等参与投资，积极落实与大型央企、自治区重点企业的战略合作。推动以钦州为基地建立中国－东盟港口城市合作机制，构筑参与中国—东盟海上互联互通合作新平台。开展“三千行动”、“大走访”活动，做好大兑现和帮办服务，进一步优化投资软环境。健全重大项目预评估机制，加强税收效益评估，提高招商引资质量。

巩固提升对外贸易水平。充分发挥背靠西南、面向东盟的区位优势，进一步扩大对外贸易规模。抓好出口基地和品牌建设，重点建设电子产品、磷酸、编织工艺品、茧丝绸等出口基地。大力发展加工贸易，扩大原油、成品油、皮革、鱼类资源等进出口。争取国家尽快批准原油非国营贸易进口资质。利用好边贸优惠政策，扶持企业开展边境贸易。积极开拓新兴市场，组织企业参加国内外知名展会和贸易洽谈会。

（六）以实施“文化建设年”为重点，在塑造城市灵魂、弘扬时代精神上求突破。扎实推动文化大繁荣大发展，激发文化创造力，增强文化竞争力，提升文化软实力，加快文化资源优势向经济优势转变。

加强社会文明教育和文化传承。积极践行社会主义核心价值观，加强“四德教育”，努力提高社会文明程度。统筹城乡精神文明建设，大力弘扬新时期钦州精神，凝聚赶超跨越的精神力量。加强历史文化的挖掘和宣传，珍视老街骑楼建筑群的文化价值，抓好钦州旧城保护管理，启动改造一至五马路旧城区、中山公园。强化文艺精品创作和文化遗产保护，保护和发展好采茶戏、跳岭头等非物质文化遗产。加大“扫黄打非”力度，加强文化市场建设和监管，推动文化繁荣发展。

大力发展文化产业。研究出台扶持文化产业发展的政策措施，培育壮大一批文化企业，发展文化创意等新兴文化业态。把坭兴陶产业作为支柱文化产业加以培育，加快建设千年古陶城、坭兴陶博物馆，加快人才培育和资本集中，促进坭兴陶企业加速发展。积极推进文化艺术单位改制，扶持演艺公司向市场化、产业化发展。打造陶艺文化节、蚝情节等节庆文化品牌。深入挖掘海洋文化，开发海洋文化精品。

完善文化基础设施和公共文化服务网络。开工建设广西北部湾博物馆、刘永福纪念馆、广播影视中心等重大项目。完善镇村文化基础设施，加强村级公共服务中心规范化建设。开展城区24小时自助图书馆试点，扶持建设镇村社区数字图书分馆。整合利用基层文化设施，组织开展文艺下乡活动。开展国家级公共文化服务体系示范县（区）申报。

积极发展文化旅游。抓住高铁开通和北部湾经济一体化的新机遇，加快建设旅游产业发展高地。实施“旅游道路畅通工程”，加快三娘湾旅游度假区、茅尾海国家海洋公园、林湖公园等景点景区开发。大力发展滨海风情游、山水田园休闲游等特色生态旅游。开工建设三娘湾中华白海豚研究保护中心、三娘湾文化旅游港、佛教文化主题公园等项目。加大文化旅游资源整合开发，积极策划推介精品旅游路线。加强与东盟国家文化、旅游产业的交流合作。加快打造海边狂欢广场、水上运动乐园等项目，多形式开展群众性文体活动。

（七）以城乡生态环境建设为重点，在建设美丽钦州上求突破。坚持促进发展与保护环境并重，大力发展绿色经济、循环经济和低碳经济，努力打响钦州绿色发展的城市品牌。

加强海洋环境保护。开展海洋生态环境调查，探索多部门联动保护海洋环境新机制。落实海岛保护规划和养殖用海规划，启动国家级海洋公园和三娘湾海洋生态文明示范区建设。切实加强白海豚保护工作，突出抓好大风江流域及三娘湾海域环境监测，减少生产经营活动对海洋环境的影响，努力实现白海豚与大工业同在。加快茅尾海综合整治，开展海域生态岸线、海岛整治和沙滩修复，实施防灾减灾工程。加大打击非法用海行为，规范用海秩序，维护海洋生态平衡。

强化节能减排。推进国家级循环化改造试点园区建设，加快园区工业管廊、污水收集管网等项目建设。加强重点节能减排工程建设，组织实施国投钦州发电公司能源系统优化项目等节能技改项目。创新工业用地模式，提高工业用地使用效率。深入推进可再生能源示范城市建设，开展星级绿色建筑示范，推广太阳能光电建筑一体化技术。推进工业、公共机构等领域的合同能源管理。强化节能减排目标管理和日常监管，开展pm2.5监测工作。普及节能知识和技术，倡导绿色低碳生活方式。

加强城乡环境治理和生态建设。加快推进钦江流域综合治理，推进农村环境连片整治和生态文明示范工程。抓好市区饮用水源保护区和备用水源建设。加快建设大榄坪污水处理厂，完善河东污水处理厂管网，提高污水处理负荷。建成运营浦北生活垃圾填埋场，建设重点镇污水处理项目，抓好镇级垃圾中转站建设。深化制糖、造纸、淀粉等行业废水治理。推进农业源规模化养殖业减排。加大森林保护和城乡绿化力度，力争森林覆盖率达到53.8%。严格土地管理制度，建立和健全执法监察共同责任制，落实耕地保护政策，健全依法用地长效机制。

（八）以保障和改善民生为重点，在维护社会和谐稳定上求突破。继续加大民生投入，统筹推进社会事业发展，让人民群众共享改革发展成果，增强人民群众的幸福感和满意度。

提高就业和社会保障水平。健全社会保险、社会救助、社会福利等社会保障体系，进一步扩大城镇职工基本养老保险、城镇基本医疗保险、工伤保险、失业保险、生育保险的覆盖面，新农保参保人数达到100万人以上。加强对农民工、被征地农民等特殊群体的就业创业帮扶，促进社会充分就业。支持劳动密集型和小微型企业发展，发展微型企业1000家，新增农村劳动力转移就业5万人。完善社会救助体系，加大城乡低保救助力度，实现动态下的应保尽保，应退尽退。加快保障性安居工程建设，新开工建设保障性住房5110套，新增发放廉租住房租赁补贴790户，推进农村危房改造，改善群众居住条件。

协调发展各项社会事业。加快北部湾大学一期工程建设，启动市商贸学校搬迁项目，抓好广西财贸职业学院和灵山、浦北教育集中区规划建设。建成市四中新校区及市三中体育馆、图书馆等项目。深入实施学前教育三年行动计划，加快建设一批城区幼儿园和150所农村幼儿园。促进义务教育均衡发展，抓好高中新课程改革，积极推进素质教育。加强教师队伍建设，培养培训一批中小学教师，聘用1000名“特岗教师”到农村中小学任教。全面落实各项资助政策，保障进城务工人员子女接受义务教育，做好农村留守儿童帮扶教育工作。实施人才强市战略，建成北部湾国际人才创业基地。深化医药卫生体制改革，抓好城乡居民大病保险试点，稳妥推进县级公立医院综合改革。加快第二人民医院扩建工程、中医医院迁建等市级医院和社区卫生服务中心、乡镇卫生院标准化建设，开展市中西医结合医院、市儿童医院等项目前期工作。继续巩固、扩大基本药物制度实施范围。实施质量强市战略，加快质检中心建设。全面做好人口计生工作，提高人口素质。落实优抚安置政策，支持部队和国防建设，深入开展双拥共建活动。贯彻国家全民健身计划，完善全民健身公共服务体系。支持工会、共青团、妇联等人民团体工作，进一步加强民族、宗教、外事、侨务、科普、新闻出版、统计、人防、海防、地震、气象、水文、档案、地方志、老龄、残疾人和关心下一代等工作。

推进社会管理创新。深入开展“携手共创平安和谐家园”活动，加强社会管理综合治理，严厉打击违法犯罪行为。推进镇村综治组织网络建设，继续实施“天网”工程。加快公检法司业务技术用房等基础设施建设。支持人民法院、人民检察院依法公正独立行使职权。继续完善社会稳定风险评估和多元矛盾纠纷调处机制，努力从源头上化解社会矛盾。加强基层政权建设，逐步提高村干部工资待遇。依法规范信访秩序，畅通群众诉求渠道。推进市应急指挥中心平台系统建设，提高防范应对突发事件综合能力。加强网络管理和应对。强化食品药品安全监管和服务工作。落实安全生产责任制，突出抓好烟花爆竹、危险化学品、非煤矿山等重点行业和领域安全监管，遏制重特大事故发生。

各位代表，坚持投资向民生领域倾斜，是政府坚定不移的工作方针。今年我们将投入40亿元，继续集中力量为民办好十件实事。一是社保惠民工程。投入7.7亿元，实施城乡居民养老保险和最低生活保障、城镇居民大病保险、农村计划生育家庭爱心保险，提高城镇居民和退休人员医疗待遇，实施残疾人托养“阳光家园计划”补助，启动一批五保新村建设。二是健康惠民工程。投入12.6亿元，开展新农合、地中海贫血防治、孕前优生健康检查和治疗项目，强化艾滋病、鼻咽癌、肝癌等疾病防治，加强社区卫生服务中心标准化建设，推进农村改厕。三是教育惠民工程。投入2.6亿元，实施职业教育、扩大农村学前教育资源、农村义务教育学校建设、学生资助等项目，开展农民工培训就业专项行动。四是强基惠民工程。投入3.8亿元，实施农村安全饮水、农田水利建设、小型病险水库除险加固、中小河流治理、水库移民新村建设、农村便民候车亭、农网升级改造等项目。五是安居惠民工程。投入5.3亿元，实施城镇保障性安居工程、农村危房改造、便民市政设施建设、城乡农贸市场改造、“天网”三期工程等项目。六是土地整治惠民工程。投入1.9亿元，实施农业综合开发土地治理、整村推进土地整治和地质灾害防治等项目。七是农补惠民工程。投入2.7亿元，实施农资综合补贴、粮食作物良种补贴、农机具购置补贴等。八是生态惠民工程。投入0.8亿元，实施森林生态效益补偿基金、“绿满八桂”造林绿化工程、农村环境连片整治、污水垃圾处理、农村户用沼气池建设等项目。九是文化惠民工程。投入0.9亿元，实施公共场馆建设、公共文化资源共享、村级公共服务中心、村级阵地“八个一”工程、村村通广播电视、电影放映、农民体育健身等项目。十是通畅惠民工程。投入1.7亿元，实施通建制村、自然村水泥路和农村屯级道路建设等项目。我们要用心办好这些实事，加快改善群众的生产生活条件，引导大家齐心协力谋发展、共奔小康富裕路。

三、加强政府自身建设

市委四届三次全会提出提前全面建成小康社会，对政府工作提出了更高要求。“空谈误国，实干兴邦”。我们要深入学习党的十八大精神，认真贯彻落实中央关于改进工作作风、密切联系群众的八项规定，切实增强赶超跨越的紧迫感和责任感，全面加强自身建设，努力建设亲民、高效、清廉、法治的政府。

（一）心系群众，建设亲民政府。始终把人民群众的需求作为政府工作的第一信号，把人民群众的满意作为政府工作的第一标准，主动深入基层、深入群众、深入实践一线，坚持问政于民、问计于民、问需于民。加强和改进信访工作，建立健全群众诉求快速处理、限时回复机制，继续优化市长服务热线、政府门户网站等信息服务平台，切实解决好群众合理诉求。

（二）改进作风，建设高效政府。坚持实干苦干，把心思花在干事业上、把精力放在谋发展上、把功夫用在解难题上。进一步加强机关作风建设，切实改进文风、会风，克服形式主义、官僚主义。继续推进政府管理创新，强化重大事项和重点工程的政务督查和效能监察，着重在招商引资、项目建设、征地搬迁等方面提升执行力。推进网上审批，优化电子政务建设，完善oa政务办公体系。强化绩效考核，完善平衡计分卡绩效考评制度，激励创先争优。

（三）勤政廉政，建设清廉政府。全面推进惩治和预防腐败体系建设，严格执行党风廉政建设责任制。扩大政府预算公开范围，严格控制“三公”经费，推进“公务卡”扩面，努力降低行政成本。严格实行财政投资评审，加强对重点领域、关键岗位、重大审批行为的行政监察和审计监督，加快公共资源交易中心建设，塑造风清气正的良好环境。

（四）依法行政，建设法治政府。全面落实行政执法责任制，完善执法流程，健全行政调解、行政复议和行政应诉机制。自觉接受人大依法监督、政协民主监督和社会监督，主动听取各民主党派、工商联、无党派人士和人民团体的意见和建议。认真办理人大代表建议和政协提案，着力提高办结满意率。深入开展“六五”普法教育，提升全民法制意识，提高领导干部依法行政能力。完善政府新闻发布制度，全面推行行政权力网上运行。

各位代表，回顾过去，我们倍感自豪；展望未来，我们信心满怀。使命和目标激励着我们，责任和重托鞭策着我们，困难和挑战考验着我们。让我们高举中国特色社会主义伟大旗帜，在市委的坚强领导下，凝心聚力，乘势而上，奋发进取，全面完成今年各项任务，为实现提前全面建成小康社会而努力奋斗!
